# Vomiting and Dysphagia Predict Delayed Gastric Emptying in Diabetic and Nondiabetic Subjects

**DOI:** 10.1155/2014/294032

**Published:** 2014-05-11

**Authors:** Doron Boltin, Ibrahim Zvidi, Adam Steinmetz, Hanna Bernstine, David Groshar, Yuval Nardi, Mona Boaz, Yaron Niv, Ram Dickman

**Affiliations:** ^1^Department of Gastroenterology, Rabin Medical Center, Beilinson Hospital, 39 Jabotinski Street 49100, Petach Tikva, Israel; ^2^Department of Nuclear Medicine, Rabin Medical Center, Beilinson Hospital, Israel; ^3^Department of Biostatistics, Rabin Medical Center, Beilinson Hospital, Israel; ^4^Epidemiology Unit, Edith Wolfson Medical Center, Holon and the Sackler Faculty of Medicine, Tel Aviv University, Israel

## Abstract

*Background.* Gastroparesis is a heterogeneous disorder most often idiopathic, diabetic, or postsurgical in nature. The demographic and clinical predictors of gastroparesis in Israeli patients are poorly defined. *Methods.* During the study period we identified all adult patients who were referred to gastric emptying scintigraphy (GES) for the evaluation of dyspeptic symptoms. Of those, 193 patients who were referred to GES from our institution were retrospectively identified (76 (39%) males, mean age 60.2 ± 15.6 years). Subjects were grouped according to gastric half-emptying times (gastric *T*_1/2_). Demographic and clinical data were extracted from electronic medical records or by a phone interview. *Key Results.* Gastric emptying half-times were normal (gastric *T*_1/2_ 0–99 min) in 101 patients, abnormal (gastric *T*_1/2_ 100–299 min) in 67 patients, and grossly abnormal (gastric *T*_1/2_ ≥ 300 min) in 25 patients. Vomiting and dysphagia, but neither early satiety nor bloating, correlated with delayed gastric emptying. Diabetes was associated with grossly abnormal gastric *T*_1/2_. Idiopathic gastroparesis was associated with a younger age at GES. No correlation was observed between gastric *T*_1/2_ values and gender, smoking, *H. pylori* infection, HBA1C, or microvascular complication of diabetes. *Conclusions Inferences.* Vomiting and dysphagia are predictive of delayed gastric emptying in both diabetic and nondiabetic subjects. Diabetes is associated with more severe gastroparesis.

## 1. Introduction

Gastroparesis is a condition of impaired gastric emptying without evidence of gastric outlet obstruction. This disorder is characterized by a poor quality of life and nutritional deficits and is associated with symptoms such as nausea, vomiting, postprandial fullness, early satiety, and bloating [1–3]. Gastroparesis is a heterogeneous disorder most often idiopathic, diabetic, or postsurgical in nature, affecting up to 1.8% of the population. Diabetic gastroparesis (DG) affects patients with long-standing diabetes mellitus usually complicated by retinopathy, neuropathy, and nephropathy [1]. The clinical and histopathological features of idiopathic gastroparesis are variable and poorly defined. For example,* H. pylori* infection has been reported to increase [4], decrease [5, 6], or not influence [7–10] the likelihood of delayed gastric emptying. Idiopathic gastroparesis has been described predominantly in young female patients with low-normal body mass [11]. The predictive value of dyspeptic symptoms is also the subject of ongoing study [11–17]. Recently, a Gastroparesis Cardinal Symptom Index (GCSI) has been developed as a valid tool for symptom stratification and for the evaluation of treatment response [3, 18]. Nevertheless, precise clinical correlates of gastroparesis remain elusive. In the first study of its sort from our geographical region, we attempt to further define the predictors of delayed gastric emptying in patients undergoing gastric emptying scintigraphy (GES) at our tertiary referral center.

## 2. Methods

### 2.1. Patients

This single-center study was conducted in accordance with the principles of the Declaration of Helsinki and Good Clinical Practice (GCP) and was approved by the Human Subjects Protection Program of the Rabin Medical Center (RMC). Dyspeptic patients undergoing GES at our institution between January 2003 and December 2009 were retrospectively identified using an established computerized chart. Only patients examined at the gastroenterology outpatient clinic and referred to GES by a gastroenterologist were included. Similarly, only subjects who had undergone upper gastrointestinal endoscopy within 1 year of GES were included. This was to ensure that no patients had gastric outlet obstruction as a cause of their symptoms. The following cases were excluded: patients with established gastroparesis undergoing follow-up GES, cases lacking* H. pylori* testing by histology, rapid urease test, or C-13 urea breath test within 3 months of GES, gastric outlet obstruction, active malignancy, pregnancy, age below 18 years, and incomplete medical records.

### 2.2. Data Collection

The following parameters were obtained from the patients' electronic record: age, sex, symptoms (dysphagia, early satiety, nausea, vomiting, bloating, abdominal pain, heartburn, and regurgitation), smoking, other active health problems including ischemic heart disease, gastroesophageal reflux disease, rheumatologic disease including scleroderma, endocrine disease including diabetes mellitus (noting microvascular complications), and thyroid disease, current medications including antireflux, opioid analgesics, and promotility agents. The electronic records retrieved included admission data, clinic visits, billing claims data, ICD-9 diagnoses registered in the centralized database, and pharmacy claims. All data were obtained by two independent reviewers (Doron Boltin and Ibrahim Zvidi). Missing parameters were obtained by a phone interview (Ibrahim Zvidi).

### 2.3. Gastric Empting Scintigraphy

Following a 14-hour fast patients received a standard 250 kca meal consisting of an egg fried in 5 g margarine, 2 slices of white bread, and 200 mL of water (15 g proteins, 26 g carbohydrates, and 9 g fat). Isotope labeling was performed by adding 1 mCi of ^99m^Tc-sulphur colloid to the egg white. Fixation of the tracer to the solid phase (necessary for measuring gastric emptying of solids) was accomplished by dissolving the isotopes inside the egg and solidifying the egg. Sequential-conjugated anterior-posterior view scintigrams of the epigastric area were acquired in a sitting position on a dual head gamma camera (Milennium VG and Infinia, GE, Buckinghamshire, UK, and E.cam, Siemens, Buckinghamshire, UK) at 30, 60, and 120 minutes following ingestion of the standardized test meal. After 2008, delayed scans were performed at 180 and 240 minutes in accordance with guidelines published at that time [19].

### 2.4. Data Analysis

To analyze the scintigraphy results the gastric region of interest (ROI) was manually drawn around the stomach on the frames at the beginning of the dynamic scan. A time-activity curve was generated from the ROI and was corrected for radioisotope decay. A linear fit of the time activity curve was used to calculate the gastric emptying half-time (gastric *T*_1/2_). A gastric *T*_1/2_ greater than 100 minutes was considered abnormal. Gastric retention, a recently introduced method to define gastric emptying rate, is considered by most authors as a better method to assess gastric emptying rate (GER). Gastric retention greater than 10% after 4 hours is considered abnormal [19]. In this study, during GES we were able to collect both gastric *T*_1/2_ and gastric retention values. However, assessment of gastric retention of tracer was feasible only after 2008 and for this reason we used gastric *T*_1/2_ for statistical analyses.

### 2.5. Statistical Analysis

Due to the presence of incalculable *T*_1/2_ values for gastric emptying (excessively prolonged) data was categorized into 3 groups as opposed to a continuum. Univariate analysis was performed for each of the acquired variables after sorting patients according to their* H. pylori* status and gastric emptying. Individual and cumulative scores were expressed as mean ± 1SD. Student's *t* test was used for continuous variables including age. Categorical variables including sex, clinical diagnosis,* H. pylori* infection, and *T*_1/2_ were analyzed with Pearson's *χ* ^2^ test and Fischer exact test. Mann-Whitney *U* test was used for symptom scores. As dependent variables we considered either individual symptom scores or the cumulative scores of symptoms. All possible cutoff values of the scores for these dichotomous variables were considered in the analysis. *P* values of 0.10 and 0.15 were chosen as cutoff points to enter and exit the stepwise procedure. Odds ratios (OR) with 95% confidence intervals (CI) were computed by means of *χ* ^2^ analysis only for the independent variables that entered the model. A cutoff value of ≥40 yr was chosen for age. Statistical evaluation was performed using the software package SPSS 21.0 (SPSS Inc., Chicago, IL).

## 3. Results

**Figure 1:**
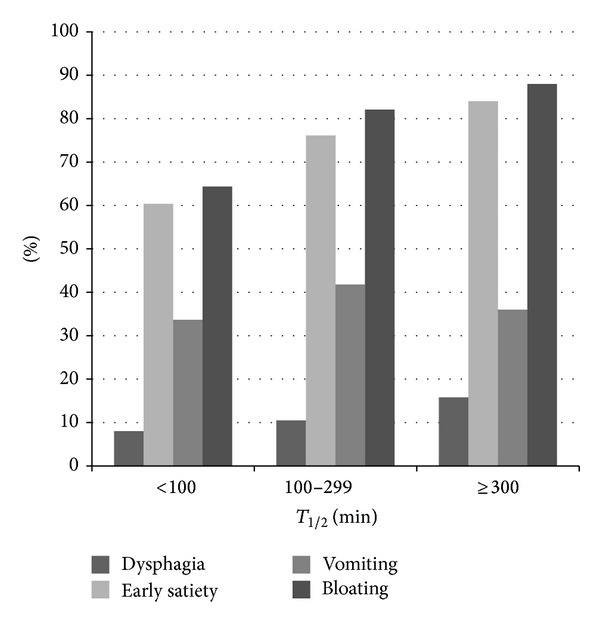
Prevalence of upper gastrointestinal tract symptoms in subjects undergoing gastric emptying scintigraphy.

**Figure 2:**
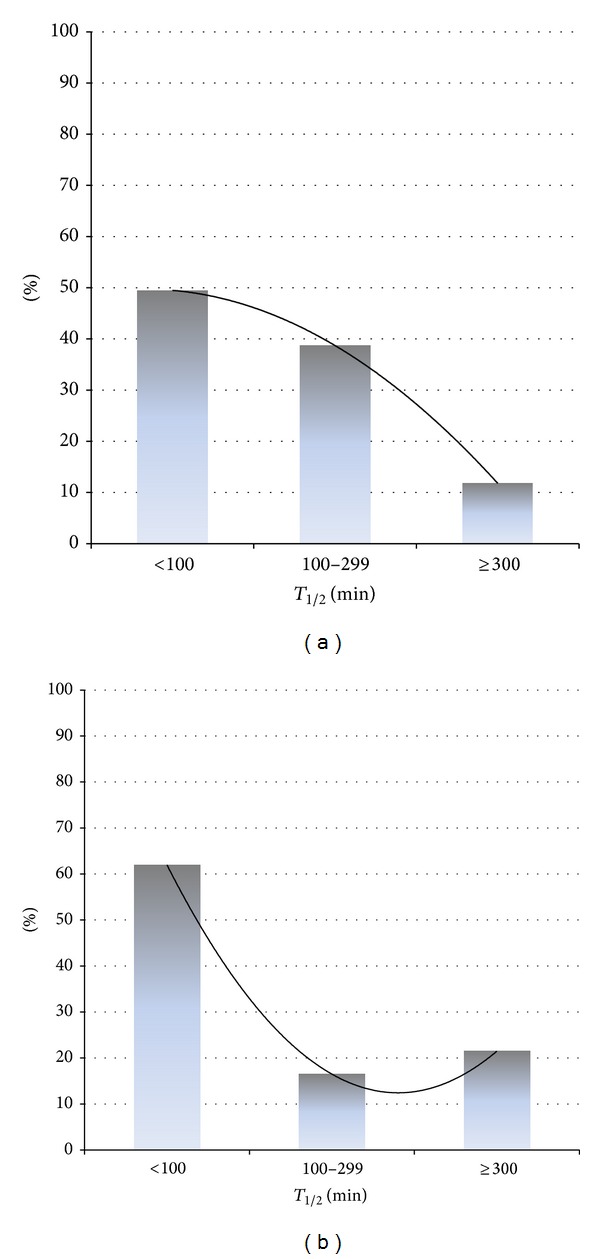
Gastric emptying in idiopathic and diabetic subjects. (a) Percentage of previously healthy (idiopathic) subjects. (b) Percentage of diabetic subjects with normal (<100 mins), mildly delayed (100–299 mins), and grossly delayed gastric emptying (≥300 mins).

During the study period, 420 dyspeptic patients underwent GES at our institution of whom 193 (46%) were eligible for inclusion in the study (76 (39%) males, mean age 60.15 ± 15.61 years). Patient characteristics are summarized in Table 1. There was a preponderance of females in all groups which did not correlate with *T*_1/2_ values. Relevant past medical history included type II diabetes mellitus in 79 patients (40.9%), scleroderma in 14 patients (7.3%), and previous esophageal/gastric surgery in 19 patients (9.8%). Evidence of* H. pylori *infection was found in 42 patients (21.8%). The *T*_1/2_ for gastric emptying was normal (0–99 minutes) in 101 patients (group 1), abnormal (100–299 minutes) in 67 patients (group 2), and excessive (≥300 minutes) in 25 patients (group 3). Significant associations between prolonged gastric emptying and patient characteristics are shown in Table 2. We found a significant association between prolonged gastric *T*_1/2_ and presenting symptoms of dysphagia or vomiting and the use of antisecretory or promotility agents (Figure 1). This was true for both patients with diabetes and those without diabetes. Diabetes was associated with significantly more excessive results (group 3) compared to group 2 (OR 1.98 (0.77–5.00; *P* = 0.03)); however, when compared to patients with normal gastric emptying (group 1), diabetes was not more common (Figure 2). There was no significant correlation between gastric *T*_1/2_ and patient age, gender, or* H. pylori* infection. Subgroup analysis demonstrated that patients with idiopathic gastroparesis were significantly younger than their counterparts with gastroparesis secondary to diabetes, surgery, or scleroderma; however, no difference in sex,* H. pylori *infection, or any other predictor was observed (Table 3).

## 4. Discussion

In the present study we describe the demographic and clinical predictors of gastroparesis in a cohort of consecutive dyspeptic patients referred for gastric scintigraphy at a single tertiary referral center. Our center is the largest hospital belonging to Clalit Health Services, the largest of five health care providers in the country with approximately 3.8 million members. This is the first such study emerging from Israel.

Of all the presenting symptoms in subjects referred for scintigraphy, only dysphagia and vomiting were independent predictors of a delayed gastric *T*_1/2_. These findings concur with Ardila-Hani et al. [12] who found positive correlation between vomiting and anorexia and delayed gastric *T*_1/2_. Interestingly, bloating was negatively correlated with gastric retention. Others have found that postprandial fullness, bloating, and pain correlate with delayed gastric emptying, but not vomiting or dysphagia [15, 20]. Ron et al. found that early satiety was the only patient-reported symptom associated with delayed gastric emptying, as assessed by breath-test [21]. Prospective studies using validated scoring systems have correlated protean symptoms with gastric retention; however, a wide variability exists [11–13, 16]. In fact, delayed gastric emptying rate (GER) is not always associated with symptoms. Indeed, gastric *T*_1/2_ is not a very specific marker of gastric dysmotility, and it has been found to be surprisingly normal in some dyspeptic patients with long-standing diabetes mellitus [21].

Dysphagia, as a predictor of gastric emptying has not been reported by other groups, is not a classic manifestation of gastroparesis and indeed is not a component of the GCSI. Our computerized charts allowed for reliable reporting of dysphagia. Nevertheless, this finding may be biased by an overrepresentation of scleroderma and upper GI surgery cases (in whom dysphagia was reported in 21%, compared to 11% in diabetic and idiopathic cases) or an overrepresentation of patients with GERD (in whom dysphagia was noted in 13.8%, compared to 9.1% without GERD). So, too, our exclusion of patients without upper GI endoscopy within 12 months of GES may have led to the overrepresentation of alarm symptoms such as dysphagia.

We found no correlation between prolonged gastric emptying and* H. pylori* infection, either as a whole group or in the subset of patients with idiopathic gastroparesis. This is in keeping with several older studies which have discounted a specific link between* H. pylori* and idiopathic gastroparesis [22, 23]. Although a minority of studies have linked* H. pylori* infection to increased or decreased gastric emptying, these studies are limited by inaccurate definitions of dyspepsia, small case numbers, nonstandardized symptom questionnaires, and methodological flaws such as the use of low-calorie test meals [5, 6, 24].

Over 40% of subjects included in this study had comorbid type II diabetes mellitus. Diabetes is a well-described cause of gastroparesis (29%) and the incidence of gastroparesis is 4.5% and 1.0% in types I and II diabetes, respectively [25]. Diabetic gastroparesis may be related to autonomic neuropathy, enteric neuropathy, interstitial cells of Cajal dysfunction, acute hyperglycemia, incretin-based medications, and altered neuroendocrine function [25]. In this study, patients with a markedly abnormal gastric *T*_1/2_ (≥300 mins) were almost twice as likely to have diabetes mellitus, when compared to subjects with lesser degrees of gastroparesis (100 mins ≤ *T*_1/2_ < 300 mins). Interestingly, a similar relationship was not observed when compared to patients with normal gastric emptying (*T*_1/2_ < 100) (Table 2). This may reflect a referral bias to scintigraphy for diabetic patients with upper gastrointestinal symptoms, leading to an overrepresentation of diabetic subjects in group 1. Alternatively, this may be a reflection of relatively well-controlled diabetes in study population (mean HbA1C was 7.7%) or preemptive treatment with promotility agents. Subanalyses did not reveal any difference in gastric *T*_1/2_ between diabetic subjects with or without microvascular complications.

Antisecretory medications were positively correlated with gastric retention. This is probably a confounding factor, simply representing a subset of patients with severe symptoms. Proton pump inhibitors and H2-receptor antagonists have no intrinsic effect on gastric emptying rate [26]. Patients receiving promotility agents had prolonged gastric emptying. This likely represents (inadequate) treatment in patients with a high pretest probability for gastroparesis. Suffice it to say, current recommendations call for stopping all medications known to affect gastric emptying for 48–72 hours; however, due to the retrospective nature of our study it is impossible to know if this was enforced.

Subgroup analysis revealed that patients with idiopathic gastroparesis were significantly younger than their counterparts with diabetes, previous surgery, or rheumatic disease. Two-thirds of subjects with idiopathic gastroparesis were female, compared to approximately half of subjects with normal gastric emptying. This is consistent with Parkman et al. who found that idiopathic gastroparesis occurs predominantly in young female patients with low-normal body mass [11, 27]. The underlying mechanism for this phenomenon is not fully understood and may be related to estrogen levels. Indeed, during the ovulatory period and pregnancy, peristalsis is decreased and constipation is commonly reported [28]. Parkman, however, described a cohort providing no comparison to other forms of gastroparesis or to subjects undergoing GES without gastroparesis.

Our study has several limitations, including the retrospective design and the absence of validated symptom questionnaires such as the GCSI. Nevertheless, every attempt was made to retrieve data on symptoms retrospectively in a thorough manner. Where missing, data was retrieved from phone interviews. All of the symptoms mentioned in Section 2 were included in the analysis and only vomiting and dysphagia were found to be independent predictors. No reliable data on body mass index (BMI) could be extracted from the electronic files. Another limitation is our inclusion of variables which may affect gastric emptying in unpredictable and unquantifiable manner. These potential confounders include GERD, prior surgery, scleroderma, and antisecretory medication. Data was partially obtained from scans performed prior to 2009 at which time a standardized test protocol (minimum 4-hour testing) was adopted [19]. For this reason extrapolated *T*_1/2_ values were used to quantify gastric emptying as opposed to the directly measurable percentage of tracer retained. This in turn precluded regarding gastric emptying as a continuous variable, as 12*T*_1/2_ values were “infinite.” Each of these factors may affect the reliability of the results. Finally, our cohort included 18 subjects with prior upper GI surgery. There is currently no data regarding what constitutes normal or abnormal gastric emptying in these patients.

In conclusion, this study identifies that vomiting and dysphagia but not bloating or early satiety are independent predictors of prolonged gastric emptying. Diabetes is associated with excessively prolonged gastric emptying. Idiopathic gastroparesis is a disease of younger women. Large well-designed prospective cohorts are needed to verify these findings.

## Figures and Tables

**Table 1:** Patient characteristics.

	*N* (%)
*N*	193 (100)
Age y (mean (SD))	60.15 (15.61)
Male	76 (39.38)
*H. pylori*	44 (22.80)
Smoking	20 (10.36)
Symptoms	
Dysphagia	25 (12.95)
Early satiety	133 (68.91)
Vomiting	71 (36.79)
Bloating	142 (73.58)
Diabetes	79 (40.93)
HBA1C^§^ % (mean (SD))	7.70 (1.87)
Neuropathy	34 (43.04)
Retinopathy	41 (51.90)
nephropathy	25 (31.65)
Antidiabetic medication^¥^	76 (96.20)
Cardiovascular disease	139 (72.02)
Hypertension	105 (54.40)
Dyslipidemia	132 (68.39)
Ischemic heart disease	43 (22.28)
Peripheral vascular disease	22 (11.40)
Scleroderma	14 (7.25)
Gastroesophageal surgery	18 (9.32)
GERD	94 (48.70)
Medications	
Oral hypoglycemic	51 (26.42)
Insulin	34 (17.62)
Antiaggregant^¶^	96 (49.74)
Antihypertensive	108 (55.96)
Antisecretory^#^	147 (76.17)
Narcotic	7 (3.63)
Metoclopramide /domperidone	57 (29.53)

^§^Data missing for 5 subjects; ^¥^insulin, oral hypoglycemic agents, or both; ^¶^aspirin, clopidogrel, or both; ^#^H2-receptor antagonist (10 cases), proton pump inhibitor (126 cases), or both (11 cases).

**Table 2:** Independent predictors of prolonged gastric emptying.

	OR (95% CI)			*P*
	Group 3/group 1*	Group 2/group 1*	Group 3/group 2*	
Dysphagia	3.44 (1.10, 10.78)	2.09 (1.05, 4.16)	1.65 (0.49, 6.00)	0.021
Vomiting	4.06 (1.14, 14.51)	2.54 (1.20, 5.35)	1.60 (0.41, 6.00)	0.008
Diabetes	0.83 (0.35, 2.01)	0.42 (0.22, 0.81)	1.98 (0.77, 5.00)	0.032
Proton pump inhibitor	0.94 (0.38, 2.35)	2.43 (1.15, 5.13)	0.39 (0.14, 1.00)	0.046
H2 antagonist	2.34 (0.78, 7.02)	0.35 (0.09, 1.28)	6.74 (1.54, 30.00)	0.002
Metoclopramide	2.43 (0.93, 6.32)	3.29 (1.64, 6.60)	0.74 (0.29, 2.00)	0.002

*Group 1: *T*_1/2_ 0–99 minutes; group 2: *T*_1/2_ 100–299 minutes; group 3: *T*_1/2_ ≥ 300 minutes.

**Table 3:** Clinical associations of gastroparesis.

	Group 1^¥^	Group 2	Group 3	Total
Idiopathic *N* (%)	46 (49.5)	36 (38.7)	11 (11.8)	93 (100)
Male *N* (%)	20 (43.5)	13 (36.1)	3 (27.3)	36 (38.7)
Age mean (SD)	59.20 (17.7)	57 (19.5)	54.64 (17.8)	57.81 (18.3)
*H. pyloriN* (%)	11 (23.9)	8 (22.2)	3 (27.3)	22 (23.7)
Secondary^§^ *N* (%)	55 (55.0)	31 (31.0)	14 (14.0)	100 (100)
Male *N* (%)	25 (45.5)	11 (35.5)	4 (28.6)	40 (40.0)
Age mean (SD)	63.45 (11.3)	59.29 (13.9)	64.57 (12.1)	62.32 (12.3)*
*H. pyloriN* (%)	11 (20.0)	5 (16.2)	6 (42.9)	22 (22.0)
Diabetes *N* (%)	49 (62.0)	13 (16.5)	17 (21.5)	79 (100)
Male *N* (%)	20 (40.8)	5 (38.5)	6 (35.3)	31 (39.2)
Age mean (SD)	63.7 (11.2)	64.2 (9.6)	64.1 (11.2)	63.8 (10.8)*
*H. pylori N* (%)	10 (20.4)	1 (7.7)	6 (35.3)	17 (21.5)

*P < 0.05 (compared to idiopathic group).

^§^Diabetes mellitus, scleroderma, and prior surgery.

^*¥*^Group 1: *T*_1/2_ 0–99 min; group 2: *T*_1/2_ 100–299 min; group 3: *T*_1/2_ ≥ 300 min.
